# Out-of-Pocket Spending on Out-Patient Care in India: Assessment and Options Based on Results from a District Level Survey

**DOI:** 10.1371/journal.pone.0166775

**Published:** 2016-11-18

**Authors:** Indrani Gupta, Samik Chowdhury, Shankar Prinja, Mayur Trivedi

**Affiliations:** 1 Professor, Institute of Economic Growth, Delhi, India; 2 Assistant Professor, Institute of Economic Growth, Delhi, India; 3 Associate Professor, Post Graduate Institute of Medical Education and Research, Chandigarh, India; 4 Associate Professor, Indian Institute of Public Health, Gandhinagar, India; Mahidol-Oxford Tropical Medicine Research Unit, THAILAND

## Abstract

Out-of-pocket spending at out-patient departments (OPD) by households is relatively less analyzed compared to hospitalization expenses in India. This paper provides new evidence on the levels and drivers of expenditure on out-patient care, as well as choice of providers, using household survey data from 8 districts in 3 states of India. Results indicate that the economically vulnerable spend more on OPD as a proportion of per capita consumption expenditure, out-patient care remains overwhelmingly private and switches of providers—while not very prevalent—is mostly towards private providers. A key result is that choice of public providers tend to lower OPD spending significantly. It indicates that an improvement in the overall quality and accessibility of government facilities still remain an important tool that should be considered in the context of financial protection.

## Introduction

High out-of-pocket spending (OOPS) is now an accepted feature of the Indian health financing system, and there is enough evidence to show that this system continues to put stress on individuals and households [1–2]. Successive National Sample Survey (NSS) data shows a steady increase in the share of OOPS in total consumption expenditure of households [3]. OOPS on hospitalization has been singled out as the most important reason for impoverishment and, therefore, India has seen the launch of a few schemes that cover hospitalization expenses of sections of population, both by the Union and the sub-national governments.

However, the role of OOPS in financing drugs and diagnostics is of recent importance, and there has been discussions around the need for making drugs and diagnostics available to the population through various additional schemes [4]. Most of the literature in India on determinants of health spending has focused on hospitalization, and there are relatively fewer separate studies on health-seeking behaviour and expenditure on outpatient departments (OPD). Nevertheless, some important findings do emerge from a review of the literature. For example, using the 60^th^ health round of the NSS, researchers find that outpatient care is more impoverishing than inpatient care in urban and rural areas alike [2].Another study shows that OOP payments on outpatient visits are mostly met through own income and savings raising the issue of ability-to-pay as a determinant of health-seeking behaviour for OPD [5]. Yet another study [6] showed that 3.5 percent of the population fell below the poverty line on account of OOPS, but this fell to 0.5 percent if OOP payments on outpatient care are excluded, with implications about the need for comprehensive coverage schemes that should include drugs or outpatient care in general.

Some disease-specific studies also confirm that the type of OPD visits—whether at private or public facilities—is an important determinant of financial burden of treatment on households [7–8]. Also, purchase of drugs and expenses on diagnostics play a key role in high OOPS and often result from OPD visits [3].

In light of these available evidences, the present study should be seen as an attempt to expand the knowledge base around the determinants of expenditure on OPD services by households—since most of the discussions have centred on hospitalization—and the need for incorporation of this knowledge in a universal health coverage (UHC) strategy. For example, while the role of private providers in hospitalization expenses is now acknowledged as a feature of the Indian health care system, relatively less attention is paid in the literature to the choice of providers for services in out-patient departments (OPD). A key feature of the present study is the level of analysis which is the district, unlike earlier studies that are based mostly on the NSS data which is representative at the state level. This paper provides fresh evidence on the level of OOPS for out-patient care based on data from 8 districts in 3 states of India. We also analyse the choice of providers for OPD visits including that of provider switches, which in turn impact on expenditure on treatment. Finally, we estimate the determinants of OOPS for OPD using a Heckman selection model, to correct for any potential bias emanating from the fact that some people have a sickness but do not seek care. Based on the results we discuss existing policy gaps in addressing the burden of OOPS from OPD in the country.

## Materials and Methods

A household survey was conducted in 8 districts in 3 states of India in 2014: Haryana (3 districts), Gujarat (3 districts) and Uttar Pradesh (UP) (2 districts) with the core objective of understanding treatment-seeking behaviour and in particular health expenditure by households and individuals. The three states were strategically selected based on economic development and health outcomes. UP is one among the 7 Empowered Action Group (EAG) states that receives particular focus in Government programmes because of their developmental challenges. Table 1 presents an overview of key indicators of these states.

**Table 1:** Key indicators of survey states.

Indicators	Haryana	Gujarat	Uttar Pradesh
Population (in millions), 2013	26	61	208
Literacy Rate, 2011	76.6	79.3	69.7
Per Capita Net State Domestic Product (INR), 2012–13	1,19,833	93,046	33,482
Annual Per Capita Public Expenditure on Health (INR), 2012–13	661	777	427
Annual Per Capita OOP Expenditure on Health (INR), 2011–12	1523	1185	1346
Population served per Government Allopathic Doctor, 2013	9503	17385	21122
Infant Mortality Rate (per 1000 live births), 2013	42	38	53
Maternal Mortality Rate (per 100,000), 2013	127	112	285

Source: Population from Population Projections for India and States 2001–2026, RGI. Literacy from Census 2011. Per capita NSDP from MOSPI. Public and private OOP health expenditure from National Health Profile-2015, CBHI.Population served per Government Allopathic Doctor from National Health Profile-2013, CBHI. IMR and MMR from SRS 2013 and 2011–13 respectively, RGI.

Note: Indian National Rupee (INR) / 1 USD: 66.4483 as on 9^th^ September, 2016.

Both Haryana and Gujarat are economically advanced states with high per capita incomes. In 2012–13 the per capita incomes at current prices in Haryana, Gujarat and UP were INR 1,19, 158, INR 96,976 and INR 33,616 respectively. In terms of health outcomes as well, Census data indicates that UP is doing poorly in terms of both IMR and MMR with values of 53 and 285, whereas Gujarat is doing much better (at 38 and 112 respectively) relative to Haryana (42 and 127 respectively) on both these indicators.

Three districts were selected from both Haryana and Gujarat while two from UP. In Haryana, 2 districts were selected that were primarily rural and urban respectively while the third district had a mixed residence of the population. In Gujarat too, the same criteria was applied for selection except that the third district viz. Chhota Udepur, was selected primarily on account of its tribal characteristics. In Uttar Pradesh, the survey was conducted in two instead of three districts due to logistical reasons.

In each district a total of 10 percent of the rural sub-centres were randomly selected as Primary Sampling Units (PSU). Similarly in the urban area, 10 percent of the urban areas enumerated for the Intensified Pulse Polio Immunization (IPPI) rounds, were sampled. In each PSU area, the sample was distributed in all the villages/colonies by probability proportional to size (PPS) method. A household enumeration survey was undertaken in each village which yielded the sampling frame. Households within each PSU were then selected using systematic random sampling. From each district, about 1500 households were selected; in all, there were a total of 12,134 households in the sample across the 3 states.

Table 2 presents the sample size for each district, share of sample that is rural and the monthly per capita consumption expenditure of the household. It indicates that Vadodara and Chhota Udepur are at the top and bottom respectively in this group based on per capita consumption expenditure.

**Table 2:** Sample characteristics of individuals.

State	District	Sample size (individuals)	Share of rural population (%)	Monthly per capita consumption expenditure (INR)
**Haryana**	Panchkula	7629	50.5	5687
	Gurgaon	7208	35.5	5437
	Panipat	7493	54.8	2506
**Gujarat**	Mehsana	7583	75.9	3359
	Vadodara	7010	41.9	6169
	Chhota Udepur	8872	85.4	1327
**Uttar Pradesh**	Kanpur Dehat	8589	91.2	3751
	Meerut	7951	53.3	5200
**All**		62, 335	62.3	4170

Data was collected using a structured questionnaire. The study (Project Number: P-614) was approved by the Institute Ethics Committee of Post Graduate Institute of Medical Education and Research (PGIMER), Chandigarh, India. Participants provided their written consent to participate in this study. The head of the household was interviewed for socio-demographic characteristics, assets and consumption expenditure. Information on education, occupation and enrolment in health insurance schemes were obtained for all individuals in the households.

Consumption expenditure comprised expenditure on food, clothing, education, utilities, transportation, purchase and construction, social ceremonies/functions.

The respondent was interviewed for any illness in last 15 days or a hospitalization during last 365 days for any member of the household, along with reason for illness or hospitalization. Reasons for illness were captured using symptoms reported by the respondent and diagnosis reported by the health care provider, in cases where care was sought. Details were elicited on the kinds of providers visited and the amount of expenditure on various items for both OPD and hospitalization. Finally, respondents were asked about the source of financing and the extent of OOPS for hospitalization episodes. In order to ensure quality, supervisors revisited 5 percent of sampled households to check accuracy of data. Any discordance was resolved through discussion and re-visiting the household.

In addition to the usual questions on how much households and individuals spent on treatment, the section on illness and treatment seeking behaviour for OPD also had an innovative section on provider switches. Essentially, patients were asked whether or not they visited more than one provider for their treatment and up to 3 such providers were probed for. This allows the analysis of the prevalence of such switches and also the reasons why individuals switch providers.

Respondents were given 15 choices for provider type that included primary health centre, community health centre, government dispensary etc. For private providers, the questionnaire included private consultant/clinic, private hospital, private pharmacy, shops etc. In addition, there was a category for charitable/missionary/NGO-run health facilities. Four categories were created based on the responses—public, private, charitable and others. Finally, the respondents were also asked about reasons for choosing a particular provider type as well as reasons for switching providers.

The econometric analysis on determinants of OOPS on OPD is based on adult respondents, with age over 15 years. To estimate the determinants of out-of-pocket expenditure, we initially considered two alternative models; the Heckman selection model [9], and the double hurdle model [10]. The Heckman model assumes that the error terms of the selection equation (whether to seek care) and the equation estimating the determinants of OPD expenditure are correlated. In other words, the decision to seek care is not entirely independent of how much care is to be sought. To estimate this model, it is necessary to satisfy the exclusion restrictions which demand that at least one variable is identified which enters the selection equation and not the equation of final interest, which in this case is the equation that estimates the determinants of OPD expenditure. There are other instances of published research papers that correct for sample selection bias to estimate determinants of OOPS [11–13]. After careful consideration of the two approaches, we decided to go for the Heckman selection model because we do believe that the decision to seek care and how much to spend are not entirely uncorrelated. The data indicated that about 16 percent of those that did not seek care did so because of financial reasons, indicating that sample selection bias cannot be ruled out.

Wherever possible, we compare our estimates with the National Sample Survey (NSS) 71^st^ Round—since the NSS health round pertains to 2014, it is comparable with the present study period. It must be reiterated that the NSS survey is not representative at the district levels, whereas the present survey is, making the district level results more robust. However, the comparisons help us benchmark our district level estimates in relation to state level estimates from NSS, to understand to some extent the degree of divergence between the two samples, and why it might be more useful to use district level data. The strength of the analysis comes from its large sample size, which also makes the results representative at the level of the districts, more so because the non-response in the survey was almost zero.

## Results

### Expenditure on OPD

The number of people reporting illness in the last 15 days was about 11 percent in the sample. The reporting varied considerably across districts, with Vadodara in Gujarat reporting less than 4 percent while Kanpur Dehat in Uttar Pradesh reporting 20 percent cases of illnesses respectively (see Table 3).

**Table 3:** OPD rates and out-of-pocket expenditures.

Districts	Proportion reporting illness, last 15 days (%)	Mean OPD expenditure in the sample (15 days) (in Rupees)	OPD expenditure per ill person (15 days) (in Rupees)	OPD expenditure as share of consumption expenditure for households with OPD visits (%)
Panchkula	14.7	107	729	9.4
Gurgaon	8.3	48	585	5.8
Panipat	9.7	35	371	6.6
**Haryana**	**10.9**	**64**	**589**	7.0
Mehsana	10.2	44	440	5.9
Vadodara	3.7	21	577	5.2
Chhota Udepur	6.4	32	536	20.7
**Gujarat**	**6.8**	**33**	**496**	12.7
Kanpur Dehat	20.0	209	1,043	15.3
Meerut	15.4	123	798	6.3
**UP**	**17.8**	**168**	**941**	13.9
**All**	**11.2**	**80**	**718**	**9.9**

The NSS 71^st^ round reports the proportion of ailing persons at any time during a 15-day reference period. This is, therefore, comparable to the estimates of morbidity in the survey. The NSS all-India figure for illness in last 15 days was 9.8 percent, with the current sample showing a mean occurrence of 11.2 percent.

Those that reported an illness were further asked about their treatment seeking behaviour, with details on OPD and hospitalization asked separately. Table 3 also reports the mean OPD expenditures for the entire sample as well as expenditure per ill person; we also report the share of OPD expenditure in total household consumption for the same period estimated for households that incurred any OPD expenditure.

Overall, the mean per capita OPD expenditure in the sample was INR 80, but the expenditure per ailing person was INR 718. There was considerable inter-district variation, with highest such expenditure being reported from Kanpur Dehat and lowest from Panipat.

Comparing the state level OPD expenditure numbers with the NSS 71^st^ round (not reported here), we find that expenditures are higher in Gujarat and UP and lower for Haryana in the present survey compared to the NSS.

Table 3 also indicates that on an average, households with an illness spent about 10 percent of their monthly consumption expenditure on OPD treatment. The share is much lower if entire sample averages are taken. As before, the variation across districts was significant, with about 21 percent in Chota Udepur and 5.2 percent in Vadodara.

**Fig 1:**
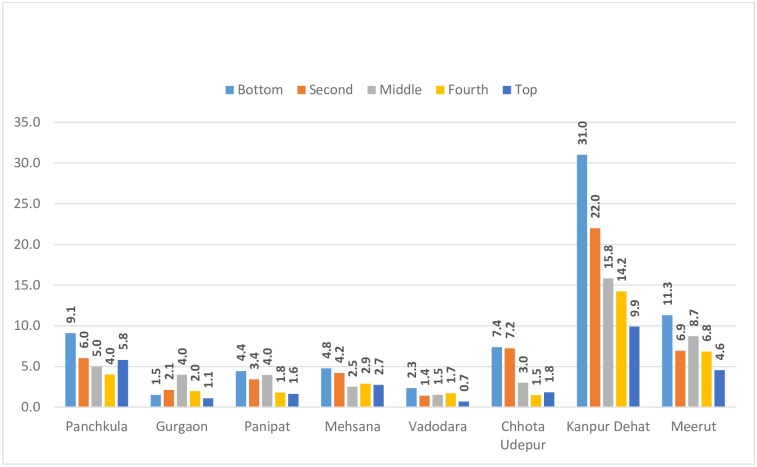
Expenditure quintile wise variation in the share (%) of OPD in total consumption expenditure.

Fig 1 shows the inter-district (and across expenditure quintile) variation in the share of OPD expenditure in total consumption expenditure for households with OPD cases. Two facts emerge from this: one, districts differ widely in terms of this share across quintiles, indicating that addressing inequities would need a district level focus. Secondly, almost all the districts –with the exception of Gurgaon—show regressivity in OPD payments though in varying degrees, with low income households paying more out of their consumption expenditure compared to those in the higher income categories.

### Choice of providers and provider switches

Overall, OPD expenditure at private providers was about 20 percent higher than expenditure incurred at government providers. However, there were significant variations across districts, with the maximum difference between private and public expenditure in Gurgaon, Mehsana, Vadodara and Chota Udepur. In some other districts like Panchkula and Panipat, however, there are hardly any differences.

In this section, in Table 4, we present the percentage distribution of OPD cases by type of provider for the first provider. Individuals accessed mainly private providers (79%), with government providers being accessed by less than 15 percent of the respondents, and almost negligible percentage choosing charitable and other providers.

**Table 4:** Selection of first provider, reasons for selection and mean distance travelled.

Facility/provider accessed	Share (%)	Main reason for accessing	Mean distance (Km)
Public	14.3	Proximity, good reputation	12.5
Private	79.0	Proximity, good reputation	7.7
Charitable/NGO	1.3	Good reputation, proximity	10.9
Others	5.4	Proximity	6.2

The main reasons for selection of either (public/private) provider were proximity and reputation, in that order. Also, the mean distance travelled differ between private and public providers: overall the private providers were much closer than the public providers as the last column indicates. An important result is that affordability did not seem to matter in choice of providers.

If one looks across districts (not shown here), the picture remains the same with maximum share of private providers, though the magnitudes differ, with Meerut reporting 90 percent and Panchkula reporting 66 percent of private providers accessed respectively. For all other districts except Panchkula, the share of private providers was over 75 percent.

Table 5 presents the main types of switches from first to second provider.

**Table 5:** Switch to second provider.

Facility/provider accessed	Share (%)
**Switch from public**	
Public to public	28
Public to private	58
Public to others	14
**Switch from private**	
Private to public	19
Private to private	72
Private to others	9

An important result is that there were not many switches; less than 5 percent of the individuals actually switched from their first selection to a second, and a negligible percentage switched from second to a third provider. A majority of the switches were from public to private or from private to private providers. For example, only 19 percent switched from private to public, whereas as many as 58 percent of those who were ill switched from a public to a private provider. However, it must be pointed out that the split between public and private providers among all those who switched did not change much from the initial distribution indicating that the preferences did not change much overall. These results—while establishing the dominance of private providers in OPD treatment in these districts, also bring out the potential that still exists in improving publicly provided services.

The main reason for switch was that the treatment did not help.

### Determinants of OPD spending: results of regression analysis

In this section, we estimate the determinants of OPD spending econometrically. The choice of variables for the regression analysis is based on similar studies reported globally. Though most of the studies were on hospitalization, the explanatory variables in OPD and hospitalization will not be too different from the perspective of the patients [14–15] [11–13].

The most common variables used to determine health expenditure among individuals are: age, gender, education, economic status, residence (rural/urban), and type of health facility visited, and we include these as well. Since the analysis is for OPD, we do not include health coverage as a predictor, since in India the existing health coverage schemes mostly cover hospitalization. In addition, and given that health expenditures are relatively higher for NCDs generally, we include a dummy for whether or not the illness was a NCD. Finally, for estimating a Heckman’s selection model, we include a duration variable in the form of number of days inactive in the selection equation (and not in the equation on determinants of OPD expenditure) on the assumption that duration of illness may influence treatment seeking behaviour and not expenditure, especially in an out-patient context. Distance travelled is not included because the OOPS already includes transport costs.

All the variables pertain to the individuals and the regressions are run at the individual level.

Table 6 presents summary statistics of the variables used in the regression analysis. It should be noted that the summary statistics pertain to those that reported an illness in the last 15 days and not the entire sample.

**Table 6:** Variables used in analysis of determinants of OOPS for OPD (means/proportions).

Variables used in the equation	Mean/proportion
**Dependent variable:** OOPS on OPD (monthly)	INR 1,436
**Independent variables**	
Per capita monthly consumption	INR 3,669
Less than 5 years of schooling or illiterate	26%
Elderly(age 60 or above)	10%
Residence rural	73%
Males	49%
Duration of illness	7.7 days
Visited government facility	12.3
Ill with an NCD	21%

Based on these variables, a regression was run, correcting for selection bias for those who were ill but did not seek care, which was about 19 percent of the ill individuals. Heckman’s selection bias model was used for this purpose, with the dependent variable being log of expenditure on OPD, converted to monthly values. The results are presented in Table 7.

**Table 7:** Determinants of out-of-pocket expenditure for out-patient care in India (Standard errors in parenthesis) (Dependent variable: log of out-of-pocket expenditure).

Independent variables	Coefficient with standard errors
Log of per capita monthly consumption	0.27*** (0.04)
Less than 5 years of schooling or illiterate among OPD patients	-0.08 (0.05)
If elderly (=1)	0.16*** (0.07)
Residence rural (=1)	0.36*** (0.06)
If male (=1)	-0.11** (0.05)
Whether visited a government doctor/facility (=1)	-0.43*** (0.07)
Whether NCD	0.73*** (0.07)
**District dummies**	
Gurgaon	0.44*** (0.11)
Panipat	-0.003 (0.10)
Mehsana	0.34*** (0.10)
Vadodara	0.44*** (0.14)
Chhota Udepur	1.1*** (0.11)
Kanpur Dehat	0.98** (0.10)
Meerut	0.79*** (0.34)
Selection equation	
Log of per capita monthly consumption	-0.02 (0.04)
Less than 5 years of schooling or illiterate among OPD patients	-0.05 (0.06)
If elderly (=1)	-0.11 (0.06)
Residence rural (=1)	0.16*** (0.05)
If male (=1)	-0.01 (0.05)
Duration of illness	-0.01** (0.005)
Whether NCD	-0.58*** (0.05)
**District dummies**	
Gurgaon	-0.24*** (0.08)
Panipat	0.45*** (0.09)
Mehsana	-0.24*** (0.08)
Vadodara	0.92*** (0.16)
Chhota Udepur	0.01 (0.10)
Kanpur Dehat	1.1*** (0.08)
Meerut	1.6*** (0.13)
N = 4863, Log Likelihood = -8609, Wald chi2 = 517	

Note: Standard errors in parentheses. Significance levels are: ***, **, * for 1, 5 and 10% respectively

The results indicate that there are two opposite effects on OOPS, a positive and significant income effect via consumption and a negative and significant impact of visits to government facilities. Among other results, the elderly and rural residents have higher OOPS relative to the respective reference groups. Having a NCD is significantly and positively related to OPD expenditure as well. Most of the district dummies seem significant with different signs compared to the reference districts, indicating strong presence of district level factors that might influence OOPS.

The selection equation is quite revealing of the treatment seeking behaviour of the sample population as well: rural residents and those with higher inactive days due to illness tend to seek care and those with NCDs seem to seek care less than those without an NCD.

## Discussion and Conclusion

The paper looked at OPD expenditure and its determinants using district level data from 3 states of India. There are four key results: the first is that the economically vulnerable individuals spend more on OPD as a proportion of per capita consumption expenditure. The second is that out-patient care remains overwhelmingly private, with concomitant impact on households, especially the more vulnerable ones. Third, generally individuals do not switch providers, but when they do, the tilt remains towards private providers, though there seems to be a reverse preference for public providers as well, if treatment by the private provider has been unsatisfactory. Finally, treatment at government facilities or providers does tend to lower OPD significantly indicating that government care is still relatively more affordable compared to private care. Other results indicate significant district level variations in OPD expenditure as well as their determinants, indicating that regional factors do play an important part in care seeking. Also, while not articulated as a reason for the respondents’ choice of providers, the average distance travelled for private providers was found to be less than the average distance travelled to reach public providers. This raises the possibility of some respondents choosing public providers, had these been closer to their residences.

The results have significant implications. Most of the discussion in India in the recent past has centred around the possibility of expanding health protection schemes for hospitalization. It is clear that schemes that do not take into account the fact that OPD is a significant part of an individual’s treatment profile—especially with increasing NCDs that result in chronic conditions requiring frequent visits—would remain ineffective as a tool for alleviating the economic impact of OOPS, especially for the poor.

Secondly, the results seem to indicate that individuals select providers based on accessibility and reputation for both types of providers (Table 4) but clearly private providers score over public providers in accessibility. Proximity as an important determinant of provider choice is also corroborated by other researchers [16]. It is now a given fact that in India, private care is a continuum ranging from traditional healers to expensive private facilities, with concomitant variation in quality. Thus, the choice of private providers mainly for OPD treatment has implications about outcomes of such treatment, especially in the almost complete absence of regulation.

The other important implication is about public providers: while relatively less preferred vis-à-vis the private, it lowers OOPS holding other parameters constant, as indicated by the regression results. Individuals also do switch from private to public providers indicating the possibility of using this as a viable argument for offering affordable and quality care through public institutions.

The debate and discussions around UHC often moves us away from a fundamental choice that India still needs to make: whether to improve the public health facilities which in principle offer to patients relatively inexpensive services compared to private providers, or offer insurance to households so that private providers become more affordable. This is very relevant in case of OPD, because currently OPD services are not covered by most health coverage and insurance schemes in the country. The latest National Health Accounts of India 2013–14 indicate that if we look at the distribution of current health expenditures according to healthcare functions, outpatient care comprises about 46 percent of the total health expenditure. Clearly, this is an area that needs to be urgently focused on in any programme that addresses health care affordability and accessibility issues. In fact, improving accessibility, affordability and quality of government facilities is the most direct way of improving OPD treatment-seeking behaviour [17–18]. This will also take care of inequities in treatment-seeking behaviour because the relatively lower cost of care would encourage those with lower ability to pay to visit public facilities.

Improving public health facilities is not an easy task, because it entails detailed planning and spending on personnel, infrastructure, drugs and other similar items. There are some instances of political commitment in selected states like Tamil Nadu and Odisha that have resulted in improved equity through improved health systems reforms focused on government facilities and interventions [19–20]. Our results, for example, show that while private providers remain the majority choice in all the districts, there is still some variation, with Panchkula in Haryana showing the highest choice of public providers at 21 percent. It may be recalled that the expenditures differences between private and government providers was also very small in this district. It is important to, therefore, understand, what kind of factors lead to choice of providers in health care, including OPD care and why some districts are doing relatively much better in being able to provide more services through public providers.

Finally, provider (public/private) preference in treatment seeking should ideally be a function of health outcomes rather than the OOPS incurred at each type of facility. While this aspect was outside the scope of this study, this work can be expanded to include health outcomes and link them with choice of providers and OOPS.

## Supporting Information

S1 Data Survey data relevant to the article, in STATA format. (DTA)
